# The Work of the Rain-Drop

**Published:** 1892-01

**Authors:** 


### THE WORK OF THE RAIN-DROP.

As every student of geology knows, the surface of the earth has undergone, in past ages, and is even undergoing at present, the most radical changes in form and condition. From the time when the earth had sufficiently cooled to allow the aqueous vapor in the air to condense and cover its surface with oceans of boiling water, change has succeeded change without cessation, although so slowly that the period of human history is too short to show any notable effect, and we are obliged to look to the records of past geological ages, in the rocks and minerals hid beneath the surface to fully appreciate the results of the forces which have acted in times gone by. Mountain ranges higher than any at present existing have been raised up only to be worn down into sand, gravel, and clay, and spread out over the bottoms of ancient oceans, which in their turn have been raised up into dry land. Volcanoes have appeared in various parts of the world, covering large tracts of country with lava, igneous rock, and volcanic *débris*. Immense fresh water swamps filled with luxuriant tropical vegetation have been transformed into the coal beds of the present day ; beds of sedimentary deposits have been covered up by subsequent layers, and baked and transformed by the internal heat of the earth into rocks that can hardly be distinguished from the primeval crust of the earth ; while glaciers, and ice-fields have now and again crept over the land, destroying all forms of life, and changing a country covered with luxuriant vegetation into an icy waste like that of Greenland at the present day.

Various geological forces have played their part in these transformations; the shrinking of the earth's crust, and the volcanic forces beneath have caused the land to rise and fall above or below the sea level ; the astronomical motions of the earth in its annual journey around the sun have caused the climate to change from time to time ; and perhaps even changes in the radiant energy of the sun itself have not been without their effect upon the earth. But above all other agençies, the little rain-drop, insignificant by itself, but mighty in numbers, has accomplished more than any other agency in the successive transformations which the face of the earth has undergone. A single rain-drop falling upon a mountain top produces no appreciable effect, but it nevertheless does something. It may move a grain of sand from its place, or even set a pebble in motion towards the valley below ; and the countless millions of rain-drops which follow it in succeeding ages, will finally succeed in wearing the mountain top away. As they roll down the mountain side, they gather into rivulets, brooks, and torrents, and their erosive force becomes greatly increased by gravitation. Another drop may fall into a crevice in the rock, there to remain until it expands in the act of freezing, and splits off a fragment, which in its turn is carried down to lower levels. Or, perhaps, the rain-drop falls as a snow-flake, which, with many others, is in time consolidated into an ice-field and glacier, which ploughs down the mountain side, crushing and grinding the rocks as it goes, into a form which can readily be transported to lower levels by the streams of water formed by its melting.

Or perhaps the rain-drop falls upon a limestone formation. A little—a very little, but still a definite quantity—of the hard rock is dissolved and carried away to the ocean, or deposited in a distant locality by the evaporation of the water. The amount dissolved by a single rain-drop is almost infinitesimal, but the great caverns found in all parts of the world owe their origin to nothing but this solvent power of innumerable drops of water. Even the hard, enduring granite will finally crumble away, one of its constituent minerals, feldspar, being decomposed and changed into clay. The erosive power of running water and the chemical action of still water supplement and aid each other in their great work.

That the streams and rivers of the more level districts are continually lowering the level of the land is evident to every one. The bars which are formed at the mouths of rivers are only the earth which has been washed down from higher regions by the rushing rain-drops, and the level of the land is constantly being lowered, and, in many cases, perceptibly so by this means. If any proof were needed of the action of this force in past times it would be found in the fossilized rain-prints, where the impressions made in soft mud by the drops of some ancient storm have been hardened into stone, and preserved to us so perfectly, that we can tell from what direction the wind was blowing in that shower of millions of years ago.

There is still another indirect way in which the rain-drop takes part in the action of geological forces. One would hardly associate the presence of water with a volcanic eruption; but water is always present and doubtless plays a most important part in the phenomena of volcanic activity. Volcanoes are always found near the sea-coast, and the presence of immense volumes of steam is always observed during an eruption. Contrary to the popular idea, neither flame nor smoke proceeds from the crater of a volcano, but only quantities of melted mineral matter, or lava, and immense volumes of steam. It was this pasty mass of finely pulverized lava—incorrectly called ashes—and water which overwhelmed the city of Pompeii and sealed it up for nearly two thousand years. Actual fire played a very small part in the destruction of that ill-fated city. There can be no doubt that water and the internal heat of the earth form a combination which will explain nearly all manifestations of volcanic forces; and so the rain-drop is shown to play another part in the geological transformations.

In short, we may say that every particle of matter above the sea level, except in regions where rain never falls, is constantly moving, slowly, but surely, towards the ocean, and has been ever since the land was first raised above the water. In past ages, the action of subterranean forces has raised the ocean beds and turned them into dry land, hills, and mountains, only to be once more returned to their native element. We cannot tell if these upheavals are to be repeated in future ages, but it seems more than probable. If not, then it is only a question of time—albeit an almost infinitely long time—when the surface of the earth will become of one uniform level, covered with a shallow ocean, and, in the polar regions, surmounted by caps of everlasting ice and snow.

But the rain-drop is not the first cause of all the changes which we have noted. When resting in the ocean it is powerless. To perform its work it must be raised up and transferred to the hillsides and mountain tops. Like all other forces on the earth, the force of the falling rain-drop and the rushing torrent has its source in the sun. The geologist can see in imagination the mountains and hills dwindling away and following the lower lands into the sea; but not a rain-drop can fall, not a brook flow down the hillside, or a river down its valley, except the water is first raised from the ocean by the same force that lights and warms our houses, that propels our railroad cars and steamships, that turns the wheels of all our machinery, and without which even life itself would be impossible. From the great central body of our system a constant stream of some mysterious manifestation of Nature is flowing to the earth; and the devastating force of the cyclone, or the imperceptible fall of a snow-flake, or rain-drop, alike have their origin in that which, in our ignorance, we are obliged to term the radiant energy of the sun.—*Popular Science News.*

